# The Re-Emergence of COVID-19 in 2022 Has Affected People’s Views on Vaccines

**DOI:** 10.3390/vaccines10111974

**Published:** 2022-11-21

**Authors:** Yufei Wu, Huanjie Li, Yunshan Wang

**Affiliations:** 1 Medical Integration and Practice Center, Cheeloo College of Medicine, Shandong University, Jinan 250012, China; 2 Research Center of Basic Medicine, Jinan Central Hospital, Shandong University, Jinan 250013, China; 3 Institute of Medical Sciences, The Second Hospital of Shandong University, Jinan 250033, China

**Keywords:** SARS-CoV-2, COVID-19 vaccines, antibodies, Omicron BA.2

## Abstract

The recurrence of the COVID-19 pandemic in 2022 has had a great impact on people’s mentality, although the government has controlled it through a series of effective measures. What is noteworthy is that the public opinion on vaccines has changed significantly, and at present, the level of public’s trust in the COVID-19 vaccine is what we are concentrating on. For the current situation, new measures should be explored. Vaccines have been proven to be effective in reducing the rate of serious cases and death among infected people. However, vaccination rates still need to be improved, especially among the elderly. For people with low antibody levels, the fourth injection is recommended. Studying vaccines effective against virus mutation is the focus of future research.

## 1. Why the COVID-19 Pandemic Outbreak Frequently?

Since December 2019, the coronavirus disease 2019 (COVID-19) caused by severe acute respiratory syndrome coronavirus 2 (SARS-CoV-2), has evolved into a global pandemic [1,2,3]. Companies in developing countries mainly developed a typical inactivated vaccine for COVID-19, and clinical trials showed that these vaccines had a good safety profile and can prevent COVID-19 [1]. At present, thanks to increasing vaccination coverage through community, online, and news media campaigns, China’s cumulative reported population vaccinated against SARS-CoV-2 has covered 89.63% of the country’s total population [4]. However, most of the elderly people in China are retired at home, which reduced older people’s access to retrieve information, meaning that the vaccination rate in older adults remains low—62% of 5.8 million people aged 60 years and over have been vaccinated and only 38% have received a booster dose [5]. The older adults have limited access to obtain information about COVID-19 vaccination, and they tend to lace more trust in traditional media and family, relatives, and friends for advice on vaccination [6]. Due to China’s national conditions and safety considerations, people aged 18 to 59 were required to be the first group for vaccination initially, which might be one of the potential reasons related to the low vaccination rate of the elderly up to now.

Under effective prevention and control measures, the pandemic continued to break out in different provinces with high frequency in China. In particular, in April 2022, the scale of COVID-19 in Shanghai, China, was comparable to that of the outbreak early in Wuhan. On 26 April 2022, about 93,890 new cases were confirmed in Shanghai in just one day [5]. Many clustered cases in China are diagnosed after a long period of hidden transmission. On the one hand, due to importation, the methods and channels of importation are diversified, which brought increasing pressure from overseas; on the other hand, the vaccination rate of the population in our country is high and most of the cases that occurred were asymptomatic infections. After infection, some cases have no symptoms or late symptoms and relatively mild symptoms [7]. The humoral immunity and cellular immunity that the vaccine stimulates the body to produce act to prevent the proliferation and spread of foreign species in the body. If the immune barrier is successful, there will be mild symptoms and sometimes even no symptoms [8,9]. In addition, COVID-19 is constantly mutating, especially the current Omicron variant of the disease, which has the characteristics of strong transmission and low pathogenicity. The spread of COVID-19 and the recurrence of the pandemic in 2022, especially in Shanghai and Beijing, have had a great impact on people’s mentality, although the government has controlled it through a series of effective measures. What is noteworthy is that public opinion on vaccines has changed a lot. At present, the level of public’s trust in the COVID-19 vaccine is the focus of our attention. We are trying to find out who are the main sources causing COVID-19 vaccine hesitancy and why.

## 2. Public Trust in Vaccines Has Changed

In December 2021, we [10] conducted a public opinion survey on the booster vaccination, and the survey results showed that 366 (92.66%) of the 395 participants in the study cohort were willing to receive a third dose of the COVID-19 vaccine. Moreover, people have 84% trust in COVID-19 vaccines. 

**Figure 1:**
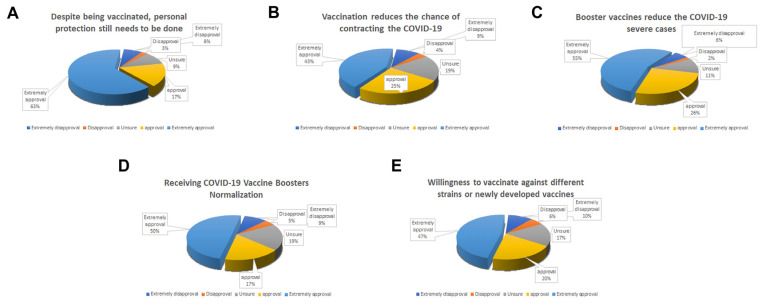
Public COVID-19 Vaccine Trust Survey. (**A**) Despite vaccinations, the public believes that personal protection is still needed. (**B**) Vaccination can reduce the chance of COVID-19 infection. (**C**) Booster vaccines reduce the number of severe COVID-19 cases. (**D**) Vaccination reduces the chance of contracting COVID-19. (**E**) Public willingness to vaccinate against different strains or newly developed vaccines. Percentages for (**A**–**E**) have been estimated using rounding, but detailed figures are mentioned in the text.

The renewed outbreak of COVID-19 in 2022 has affected people’s views on vaccines. A total of 424 volunteers were recruited from 20 May to 1 June 2022, and a questionnaire survey was conducted through WeChat mini program. The age distribution is shown in Table 1. The results of the questionnaire survey show that 6 (1.42%) of the participants had not received one shot of vaccine yet because they are hesitant about the available COVID-19 vaccine. Many people received one or two shots, probably because of their reaction to the vaccine. However, despite being vaccinated, the public still thinks that the new coronavirus is terrible and will have an impact on life. We investigated whether the respondents felt that using personal protection was important. In Figure 1A, 63.4% of the people believe that it is very necessary to do a good job of protection, 26.08% of the people think it is necessary, 9.57% of the people hold a neutral opinion, and 10.29% of the people disagreed. Thus, does this mean that people do not trust vaccines? Are they unwilling to continue with booster vaccinations? The following surveys provide the answers.

In Figure 1B, 42.58% of respondents strongly agree that vaccination will reduce the probability of contracting the SARS-CoV-2, 25.46% agree, and 18.9% hold a neutral opinion. In Figure 1C, 55.26% strongly agree that the booster injection will reduce the incidence of severe illness, 26.08% agree, 11% are neutral, and only 7% are against it. More than half of the population still believed vaccination is an effective protection measure after experiencing several outbreaks.

In Figure 1D, 49.76% strongly support the regularization of vaccination against the COVID-19, 17.22% support it, 19.14% hold a neutral attitude, and 13.88% disagreed. If there were a newly developed vaccine against a different COVID-19 strain, would the population be willing to get it? We have also investigated that question. In Figure 1E, 47.13% are very willing to be vaccinated, 20.1% support, 16.75% are neutral, and 16.04% are against it.

After the outbreak in Shanghai, the public’s trust in vaccines decreased significantly, and satisfaction in many related surveys dropped below 50%. The same was true after the outbreak in Israel, which raised questions about the efficacy of the SARS-CoV-2 BNT162b2 vaccine (Pfizer-BioNTech) with the B.1.1.529 (Omicron) mutation [11]. Why did the public’s support for vaccination drop significantly? Is the vaccine is effective or not? With the above questions, we have concluded a few interesting points.

## 3. What Should We Do in the Face of Frequent Outbreaks?

First, the increasing the number of confirmed cases put pressures on people’s psychology, which inevitably leads to doubts about the effectiveness of vaccines, and people’s willingness to get vaccinated gradually declines. A small number of people have been vaccinated against COVID-19 but still have positive nucleic acid tests or have been diagnosed with mild COVID-19. This may be the most influential and immediate cause of vaccine trust.

Second, the newly isolated SARS-CoV-2BA.2.2 subtypes in Shanghai are all SARS-CoV-2 BA.2.2 subtypes, which, have also been detected in Hong Kong, elsewhere in China, the United Kingdom, Australia and other places [12]. SARS-CoV-2 itself is prone to genetic mutation; the resulting Omicron mutant virus has increased affinity with upper respiratory cell receptors, is highly infectious, and has a shorter incubation period [6]. The antiviral effect of vaccines also has a certain impact. These factors may contribute to the occurrence of breakthrough infections. Due to the continuous mutation of the virus, the public may think that the vaccine has no protective effect against the new mutant strain, so their willingness to receive a booster vaccine is no longer strong.

Third, although Omicron BA.2 is progressively less virulent, severe outcomes and considerably higher mortality have been reported in unvaccinated populations [13]. This shows that the COVID-19 vaccine is effective against the Omicron variant, and it can reduce the fatality rate and severity rate.

Fourth, in this Shanghai epidemic, the rate of severe patients who received more than one shot of the vaccine was less than 5% [12]. It is worth noting that the mortality rate of the elderly remains high, which is related to the low vaccination rate of the elderly [14]. It is also related to the elderly themselves suffering from obesity, hyperlipidemia, hypertension, and other basic diseases [15,16]. It can be speculated that other confirmed cases and positive patients may be people who have not been fully vaccinated or have not been vaccinated at all. Although the public’s trust in vaccines has decreased, the COVID-19 vaccine is beneficial for the prevention and control of the pandemic.

Finally, vaccination rates among young people are already high (over 80%) [4,17]. According to the recent reports [18], infection with the mutant virus (Omicron) causes asymptomatic or mild fever or cough mostly in young people. There are rarely serious complications. This could be due to the weakened virulence of the mutant virus or neutralizing antibodies produced after vaccination in young people. When booster inoculation should be conducted depends on the level of the neutralizing antibody titer. Therefore, neutralizing antibody detection is very important before booster vaccine inoculation, and it was found that the antibody changes were significantly different among individuals in our follow-up study.

## 4. Conclusions

In summary, we still need the following measures: intensify the promotion of vaccination, popularize the vaccination of the whole population, and increase the vaccination rate of booster vaccine; accelerate research and development into mutant vaccines; encourage the vaccination of the elderly to increase the vaccination rate; and strengthen the detection of vaccine antibodies [19]. Multiple channels of publicity based on and supported by facts to inform the elderly, especially people with medical conditions, are still necessary. They should fully understand that vaccines can indeed reduce severe illness and address their concerns. In addition, according to China’s national conditions, most of the elderly are at home. The young people in the family need to play an important role in popular science education for the elderly so as to improve their vaccination rate. For people with low antibody levels, the fourth injection is recommended (in this regard, our research group has conducted long-term related clinical trials to confirm the necessity of detecting antibodies [20]).

## Figures and Tables

**Table 1:** Characteristics of questionnaire participants.

Group of Age	n	%	Group of Vaccination Doses	n	%
12–18	2	0.47%	1 dose	7	1.65%
19–25	108	25.47%	2 doses	86	20.28%
26–40	207	48.82%	3 doses	325	76.65%
41–60	100	23.58%	Not vaccinated	6	1.42%
over 60 years old	7	1.65%			
	424	100%		424	100%

n: number of participants; %: the percentage of the total number of participants in this group.

## Data Availability

Data analyzed in this study have been shown in the article.
